# Multivariate linear mixed model enhanced the power of identifying genome-wide association to poplar tree heights in a randomized complete block design

**DOI:** 10.1093/g3journal/jkaa053

**Published:** 2021-01-05

**Authors:** Yuhua Chen, Hainan Wu, Wenguo Yang, Wei Zhao, Chunfa Tong

**Affiliations:** 1 Key Laboratory of Forest Genetics & Biotechnology of Ministry of Education, Co-Innovation Center for Sustainable Forestry in Southern China, Nanjing Forestry University, Nanjing 210037, China; 2 School of Animal Science and Technology, Jingling Institute of Technology, Nanjing 210038, China

**Keywords:** genome-wide association study, randomized complete block design, mixed linear model, single-nucleotide polymorphism, *Populus*

## Abstract

With the advances in high-throughput sequencing technologies, it is not difficult to extract tens of thousands of single-nucleotide polymorphisms (SNPs) across many individuals in a fast and cheap way, making it possible to perform genome-wide association studies (GWAS) of quantitative traits in outbred forest trees. It is very valuable to apply traditional breeding experiments in GWAS for identifying genome variants associated with ecologically and economically important traits in *Populus*. Here, we reported a GWAS of tree height measured at multiple time points from a randomized complete block design (RCBD), which was established with clones from an F_1_ hybrid population of *Populus deltoides* and *Populus simonii*. A total of 22,670 SNPs across 172 clones in the RCBD were obtained with restriction site-associated DNA sequencing (RADseq) technology. The multivariate mixed linear model was applied by incorporating the pedigree relationship matrix of individuals to test the association of each SNP to the tree heights over 8 time points. Consequently, 41 SNPs were identified significantly associated with the tree height under the *P*-value threshold determined by Bonferroni correction at the significant level of 0.01. These SNPs were distributed on all but two chromosomes (Chr02 and Chr18) and explained the phenotypic variance ranged from 0.26% to 2.64%, amounting to 63.68% in total. Comparison with previous mapping studies for poplar height as well as the candidate genes of these detected SNPs were also investigated. We therefore showed that the application of multivariate linear mixed model to the longitudinal phenotypic data from the traditional breeding experimental design facilitated to identify far more genome-wide variants for tree height in poplar. The significant SNPs identified in this study would enhance understanding of molecular mechanism for growth traits and would accelerate marker-assisted breeding programs in *Populus*.

## Introduction

The genus *Populus* (Salicaceae) is a deciduous and dioecious tree taxon, comprising aspens, poplars, and cottonwoods, widely distributed in the Northern Hemisphere. Because of its unique biological characteristics, such as rapid growth rate, small genome size, facile asexual reproduction, and easy transgenesis, the genus has been selected as a model system in forest trees (Bradshaw *et al.* 2000). In the past three decades, poplar breeders focused on dissecting genetic architectures underlying growth traits targeted for producing new cultivars to meet the intensive need for wood-based products including timber, paper, and pulp or for bioenergy to mitigate carbon emissions (Taylor 2002). To investigate the genetic mechanism of ecologically and economically important traits, both molecular marker and phenotype data across a genetic mapping population are prerequisite to establish a statistical model for analyzing the marker-trait relationship. One of the approaches for this task is to identify quantitative trait loci (QTLs) on genome based on genetic linkage maps constructed with a set of markers generated from a full-sib family, like a backcross (BC) and F_2_ population in inbred lines or an F_1_ hybrid population in outbred species (Lander and Botstein 1989; Zeng 1994; Tong *et al.* 2012; Liu *et al.* 2017). Another approach is so-called genome-wide association study (GWAS), which allows to detect markers closely linked to QTLs with a large number of single-nucleotide polymorphisms (SNPs) in natural populations or multiple families (Gonzalez-Martinez *et al.* 2007; Li *et al.* 2014; Zhao *et al.* 2019).

In the 1990s, Bradshaw and Stettler (1995) first conducted QTL analysis of 2-year stem growth traits in an F2 hybrid population of *Populus trichocarpa* and *Populus deltoides*. Later on, Wu (1998) performed QTL mapping for 3-year growth traits in the same F2 population. Maybe due to the sparse linkage map or insufficient number of individuals used in the analyses, the two early studies were not able to detect more than two QTLs for the tree height. Recently, Monclus *et al.* (2012) identified about 7 QTLs on average controlling tree height and circumference measured in the first and the second year in an F1 hybrid population of *P. deltoides* × *P. trichocarpa*. More recently, Du *et al.* (2016) detected 3 and 6 QTLs for tree height and diameter at breast height, respectively, using a large number of progeny in an F1 population derived by crossing the female ‘YX01’ (*P*. alba × *P. glandulosa*) and the male ‘LM50’ (*P. tomentosa*). These previous studies applied the traditional molecular markers such as RAPD, RFLP, AFLP and SSR, probably limiting the power of detecting QTLs due to their low throughput (Tong *et al.* 2016). With advances in the next-generation sequencing (NGS) technologies, thousands of SNPs can be obtained across many individuals in a fast and low-cost way for QTL mapping. Recently, Tong and colleague performed a series of studies on extracting a large number of SNPs with restriction site-associated DNA sequencing (RADseq) technology, constructing genetic linkage maps, and mapping QTLs in an F1 hybrid population of *P. deltoides* and *P*. simonii (Mousavi *et al.* 2016; Tong *et al.* 2016; Liu *et al.* 2017; Yao *et al.* 2020). Meanwhile, GWAS emerged as a powerful method for identifying SNPs associated with the growth traits in poplar. Liu *et al.* (2018) conducted GWAS with 156,362 SNPs to identify significant SNP effects on the dynamic growth of tree diameter and height in a full-sib family of *P. deltoides* and *P. euramericana*.

In various GWAS, linear mixed models (LMMs) have been widely used with multiple available software packages such as TASSEL (Bradbury *et al.* 2007), EMMA (Kang *et al.* 2008), and GCTA (Yang *et al.* 2011). Because algorithms for fitting LMMs involve nonlinear optimization problem and have high computational cost (Zhou and Stephens 2014), these software packages have their own limitations in estimating genetic parameters (Zhang *et al.* 2009). It is because of this reason that two-stage approaches were applied to reduce the computational burden especially in plant GWAS, where replicated plants within blocks and plots are often used in traditional experimental designs (Xue *et al.* 2017). In some scenarios, the first stage was to obtain the best linear unbiased estimate (BLUE) or prediction (BLUP) for each line from a linear model with environmental effects but without any marker effects, whereas in the second stage the BLUE or BLUP was used as a dependent variable to perform GWAS with a reduced LMM (Zhang *et al.* 2009; Lipka *et al.* 2013; Xue *et al.* 2017; Vanous *et al.* 2018). Although most GWAS were conducted under a univariate framework, the use of multivariate linear mixed model (mvLMM) for GWAS is increasingly important because it is powerful to detect genetic variants that affect multiple traits or different growth stages (Zhou and Stephens 2014; Liu *et al.* 2018; Carlson *et al.* 2019). Like the two-stage approaches, different reduced strategies were also applied in multivariate GWAS such as using ratios between two phenotypes (Gieger *et al.* 2008) and the principal components of multiple traits (Aschard *et al.* 2014; Rice *et al.* 2020) to perform a univariate association analysis. However, from the statistical perspective, the direct use of mvLMM by incorporating various environmental effects undoubtedly enhances the power of GWAS not only over the univariate analysis but also over the reduced approaches (Galesloot *et al.* 2014; Zhou and Stephens 2014; Onogi 2019).

In this study, we reported a multivariate GWAS of tree height with longitudinal data from a randomized complete block design (RCBD). The design was established with clones from the F_1_ hybrid progeny of *P. deltoides* and *P. simonii* as described above. Each clone has several cuttings planted in different blocks, which have the same genome as a single seedling tree in the F_1_ population. In the previous studies (Mousavi *et al.* 2016; Tong *et al.* 2016), we performed RAD sequencing of many individuals in the hybrid population. By mapping RADseq data of each clone to the reference genome of *P. trichocarpa* (Tuskan *et al.* 2006), we obtained 22,667 SNPs across 172 clones. With the SNP genotype data at these SNPs for each individual, we applied mvLMM to perform GWAS of tree height measured over multiple time points using the R package EMMREML (https://cran.r-project.org/web/packages/EMMREML, accessed January 6, 2021). To compare with the multivariate method, we also conducted a univariate GWAS of the tree height at each single time point using a two-stage approach with the software TASSEL (Bradbury *et al.* 2007). The result showed that the multivariate method showed a superior ability over the univariate approach in detecting the association of SNPs with the tree height. Moreover, we could identify far more significant SNPs associated with the tree height than the previous QTL mapping studies in *Populus*. In addition, we also investigated the candidate genes of the significant SNPs, which were related to plant hormones, to the growth and development of tree tissues, and to response to stresses, or involved in photosynthesis.

## Materials and methods

### Plant materials and measurement of growth traits

An RCBD was established for testing the clones from an F_1_ hybrid population, which was derived by crossing *P. deltoides* and *P. simonii* in the springs from 2009 to 2011. The two parents have substantial differences in growth and adaptability and their hybrids display significant segregation traits in morphology and physiology (Mousavi *et al.* 2016; Tong *et al.* 2016). In the spring of 2017, a total of 234 clones were chosen to plant with 3 blocks, 6 cuttings for each clone per plot within a block, and a 50 × 60 cm spacing on Xiashu Forest Farm of Nanjing Forestry University, Jurong County, Jiangsu Province, China. During the growth season, each tree was measured in cm using a telescoping height measuring pole for height at 8 different times, that is, on June 8 (T1), June 23 (T2), July 6 (T3), July 16 (T4), July 27 (T5), August 14 (T6), September 2 (T7), and October 14 (T8), 2017. We preliminarily performed correlation analysis and multivariate analysis of variance for these phenotypic data with SAS 9.3 software (SAS Institute, Cary, USA).

### SNP genotyping

In our previous studies, we performed RADseq of hundreds of individuals in the *Populus* F_1_ hybrid population (Mousavi *et al.* 2016; Tong *et al.* 2016). Of the clones used in the RCBD, 172 clones and their two parents were sequenced previously and the RADseq data were already deposited at the SRA database in NCBI with accession numbers in Supplementary Table S1. These RADseq data were filtered to obtain high-quality (HQ) reads data using the NGS QC toolkit with default parameters (Patel and Jain 2012). We used the HQ reads data to call SNP genotypes for each clone with the reference genome of *P. trichocarpa*. The whole calling procedures were almost the same as described in our previous studies such as Mousavi *et al.* (2016) and Yao *et al.* (2020) except for the utilization of different reference sequence. In brief, the paired-end (PE) reads of each clone were first aligned to the reference sequence with the software BWA (v0.7.17) to generate a SAM formatted file (Li and Durbin 2009). The SAM file was converted into BAM formatted file which was further sorted and indexed with SAMtools (v1.9) (Li *et al.* 2009). Then, the sorted BAM file was used to generate a BCF file and further to a VCF file using the software BCFtools (v1.9) (Li *et al.* 2009). Finally, we filtered the VCF file to obtain SNP genotypes for each clone such that a heterozygous allele has a read coverage depth (DP) of at least 3 and the quality of each SNP genotype is greater than 30.

After obtaining SNP genotypes of each clone, we further filtered the genotype data across the 172 clones by considering the missing genotype rate and the segregation ratio at each SNP site. We kept those SNPs that were not seriously deviated from the Mendelian segregation ratios (*P *>* *0.01), which possibly include the ratios of 1:1, 1:2:1, and 1:1:1:1 due to the complicated genetic structure of the F_1_ hybrid population in outbred forest species (Maliepaard *et al.* 1997; Tong *et al.* 2010). Meanwhile, if there were more than 5% missing genotypes at an SNP site, it was removed from the data set.

### Statistical model for association analysis

The mvLMM was applied to find an SNP association to the tree height as follows:

(1) $$y_{\mathrm{ijkt}}=\mu_{t}+B_{it}+M_{jt}+G_{jt}+e_{\mathrm{ijkt}},$$

where $y_{\mathrm{ijkt}}$ is the height of the *k*th tree of the *j*th clone in the *i*th block at the *t*th time point, $\mu_{t}$ is the overall mean of tree height, $B_{it}$ is the effect of the *i*th block, $M_{jt}$ is the genotype effect of the *j*th clone at the tested SNP site, $G_{jt}$ is the polygene background effect of the *j*th clone (Yu *et al.* 2006), and $e_{\mathrm{ijkt}}$ is the residual effect. It is assumed that $B_{it}$ and $M_{jt}$ are fixed effects each with the sum-to-zero constraint, whereas $G_{jt}$ and $e_{\mathrm{ijkt}}$ are the random effects with $G_{jt}∼N(0,\;\sigma_{g_{t}}^{2})$ and $e_{\mathrm{ijkt}}∼N(0,\;\sigma_{e_{t}}^{2})$. In matrix form, model (1) can be expressed as:

(2) Y=BX+GZ+E,

where Y is the m×n matrix of tree heights of n individuals at the m time points; X is a p×n known design matrix of fixed effects, including overall mean, block effects, and individual genotype effects at the tested SNP site; B is the m×p matrix of fixed-effect coefficients; G is the m×c matrix of random additive genetic effects with $\mathrm{Vec}(G)\sim N_{m\times c}\left(0,\;A⊗V_{G}\right)$, where c is the number of clones and Vec denotes the matrix vectorization function (Searle *et al.* 2006, pp. 458),Ais the additive relationship matrix for the c clones and $V_{G}$is the additive genetic covariance matrix for the m time points ;Zis thec×ncoefficient matrix corresponding to the matrixG;Eis the random residual matrix with $\mathrm{Vec}(E)\sim N_{m\times n}\left(0,\;I_{n}⊗V_{E}\right)$. Hence, the covariance matrix of Vec(*Y*) can be expressed as:

(3) $$V=\mathrm{cov}\left(\left(Y\right)\right)=Z^{\prime }AZ⊗V_{G}+I_{n}⊗V_{E}.$$

Since the clones in the RCBD were from a full-sib family and their parents were unrelated and not inbred, the coefficient of additive genetic covariance between any two different clones is 0.5 in theory (Loiselle *et al.* 1995; Lynch and Walsh 1998), leading to theAmatrix with ones on the diagonal and 0.5 elsewhere.

We used the R package EMMREML to calculate the REML estimates of $V_{G}$ and $V_{E}$ and then the BLUE of B (https://cran.r-project.org/web/packages/EMMREML, accessed January 6, 2021). To test the effects of SNP genotypes, an F statistics was used under the null hypothesis *M* Vec (*B*) = 0for a full-rank q×mp matrix, as:

(4) $$F=\frac{1}{q}{\left(M\;\mathrm{Vec}(B)\right)}^{\prime }{\left[M{\left(\left(X⊗I_{t})V^{-1}(X^{\prime }⊗I_{t}\right)\right)}^{-1}M^{\prime }\right]}^{-1}\left(M\;\mathrm{Vec}(B)\right),$$

withqnumerator degrees of freedom andt(n−p)denominator degrees of freedom (Kang *et al.* 2008). The *P*-value for testing each SNP was adjusted based on Bonferroni-correction and the genome-wide false discovery rate (FDR) was set to be 0.01. As the method described in Xu (2003), the percent of phenotypic variance explained by a significant SNP was calculated as:

(5) $$R^{2}=1-\frac{RSS_{1}}{RSS_{0}},$$

where $RSS_{0}$ and $RSS_{1}$ are the residual sums of squares under the null and full hypothesis models, respectively.

To compare the multivariate GWAS approach to the univariate method, we performed GWAS of the tree height at each time point separately with two-stage approach using the software TASSEL (Bradbury *et al.* 2007). First, the best linear unbiased estimates (BLUEs) of the clone effects were obtained with the reduced linear model from model (1) by omitting the SNP genotype effects and fixing time point *t* as follows:

(6) $$y_{\mathrm{ijk}}=\mu +B_{i}+G_{j}+e_{\mathrm{ijk}}.$$

Second, the BLUEs at a fixed time point were used for the association analysis using TASSEL with parameters set as “-mlmVarCompEst P3D -mlmCompressionLevel None.”

Additionally, to obtain the heritability of the tree height at each time point, we used model (6) to first estimate the genetic and residual variance components with EMMREML and then to calculate the heritability as:

(7) $$h^{2}=\frac{\sigma_{g}^{2}}{\sigma_{g}^{2}+\sigma_{e}^{2}}.$$

### Investigation of candidate genes

The upstream and downstream genes of the significant SNPs were investigated for candidate genes affecting the tree height. If a gene harbored an SNP that had a linkage disequilibrium (LD) value ($r^{2}$) above a threshold with a neighboring significant SNP, then this gene was considered as a candidate gene for further investigation. The LD threshold was determined by performing LD decay analysis with all the SNPs using the software PopLDdecay (Zhang *et al.* 2019). After that, the coding sequences of the candidate genes around each significant SNP were extracted from the gene annotation of *P. trichocarpa* at Phytozome (v4.1; https://genome.jgi.doe.gov). These genes were annotated afresh by first performing blast searches with their coding sequences against the non-redundant protein database (Altschul *et al.* 1990; Pruitt *et al.* 2007) and then mapping the hits to GO terms with Blast2GO (https://www.blast2go.com). The result of these gene annotations was saved in a text file, in which we searched the keywords related to the tree growth and development as well as response to stresses. Furthermore, these candidate genes were used to perform GO enrichment analysis with Blast2GO for finding which GO terms are over-represented for the growth of tree height.

### Data availability

The RADseq data of the 172 clones have been deposited in the SRA database at the National Center for Biotechnology Information (NCBI) with accession numbers presented in Supplementary Table S1. The phenotypic and genotypic data generated for GWAS in this study can be found in Supplementary Tables S2 and S6, respectively. All of the supplemental materials (Supplementary Tables S1–S13 and Supplementary Figures S1 and S2) for this study are available at figshare DOI: 10.25387/g3.13017866.

## Results

### Phenotype and genotype data

**Figure 1:**
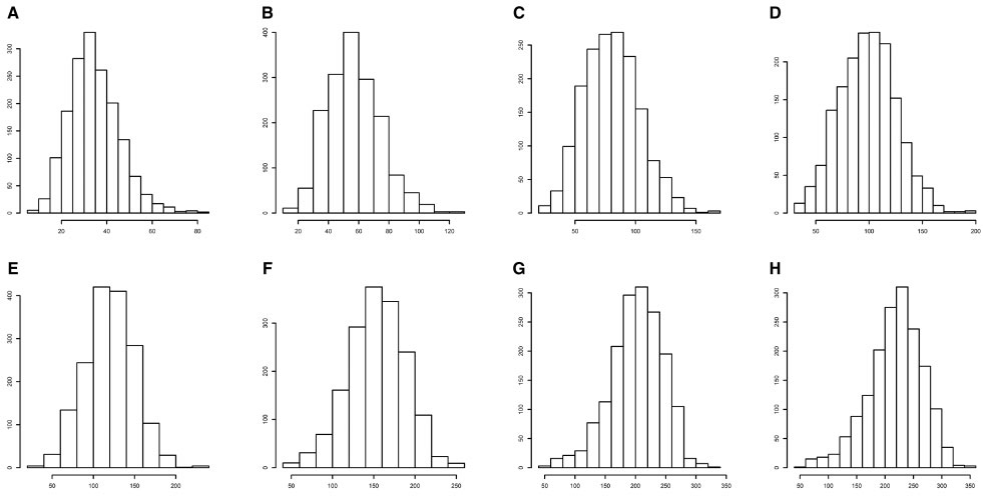
Histograms of tree height measured in the randomized complete block design at eight different time points: (A) June 8, (B) June 23, (C) July 6, (D) July 16, (E) July 27, (F) August 14, (G) September 2, and (H) October 14 in 2017.

We successfully obtained tree height and SNP genotype data of 172 clones and a total of 1664 individual trees (Supplementary Table S2). The tree heights were measured at 8 different times during the growth season in 2017. Histograms showed that the tree height basically followed a normal distribution at each time point (Figure 1). The average and standard deviation of the tree height were consistently increased across the growth season, but the coefficient of variation (CV) was decreased from about 32% to 22% (Supplementary Table S3). We also found that the CV of the tree height increment between successive time points varied smoothly in a range of 29.60–37.16% over the first six time intervals. However, the CV of the increment abruptly rose to 63.83% at the last time interval (September 2 to October 14, 2017). It is mainly due to the fact that the mean of the increment was the smallest and the standard deviation was relatively high for the last time interval (Supplementary Table S3). Meanwhile, the heritabilities at each time point did not change much with a range of 0.54–0.55 during the vigorous growth season (June to July), but then dropped to 0.47 in the late growth season (August to October; Supplementary Table S3). Correlation analysis of the phenotypic data showed that the tree height was significantly correlated between any two time points, with a coefficient of over 0.94 (*P *<* *0.01) between adjacent time points and a minimum coefficient value of 0.541 between the first and the last measure time points (Supplementary Table S4). Furthermore, we also calculated the genetic correlation coefficients of the tree heights at different time points from the genetic covariance matrix estimated with model (1) by ignoring SNP effects. The result showed that the genetic correlation coefficients were consistently higher than the phenotypic correlation coefficients and that the genetic coefficients between adjacent time points were all greater than 0.960 (Supplementary Table S4). Moreover, multivariate analysis of variance for the longitudinal data showed that the effects of tree height were significantly different among the blocks, the clones, and the interactions of blocks and clones (Supplementary Table S5). These primary statistical analyses showed that the tree height over multiple time points in the RCBD variated largely and was worth further exploring the molecular mechanism.

A total of 22,670 SNPs across the 172 clones (Supplementary Table S6) were obtained by mapping their RADseq data separately to the reference genome of *P. trichocarpa* (v4.1; https://genome.jgi.doe.gov). Each clone had an average of 16.89 million RADseq reads and 4.61 Gb data with a mean genome coverage depth of 9.6X (Supplementary Table S1). After a stringent quality control with NGS QC toolkit, an average amount of 4.45 Gb HQ reads data per clone was remained for calling SNP genotypes. Since the clones were from the F_1_ hybrid population in *Populus*, as expected, the majority of SNPs were segregated in the ratio of 1:1 (*P > *0.01) with a minority in 1:2:1 and 1:1:1:1 (Table 1). Each SNP genotype was satisfied such requirements that the allele of a heterozygous genotype had a coverage depth of at least three reads and the coverage depth for the allele of a homozygous genotype was at least 5. In addition, the quality of each genotype had a Phred score of at least 30. The missing genotype rate at each SNP was controlled to be not greater than 5%.

**Table 1:** Summary of SNPs obtained across clones in the randomized complete block design

Segregation type	Ratio	Genotype	Number
*aa*×*ab*	1:1	*aa*, *ab*	8,968
*aa*×*bc*	1:1	*ab*, *ac*	23
*ab*×*aa*	1:1	*aa*, *ab*	13,512
*ab*×*cc*	1:1	*ac*, *bc*	48
*ab*×*ab*	1:2:1	*aa*, *ab*, *bb*	105
*ab*×*ac*	1:1:1:1	*aa*, *ab*, *ac*, *bc*	14
Total			22,670

### Significant SNPs associated with tree height

**Figure 2:**
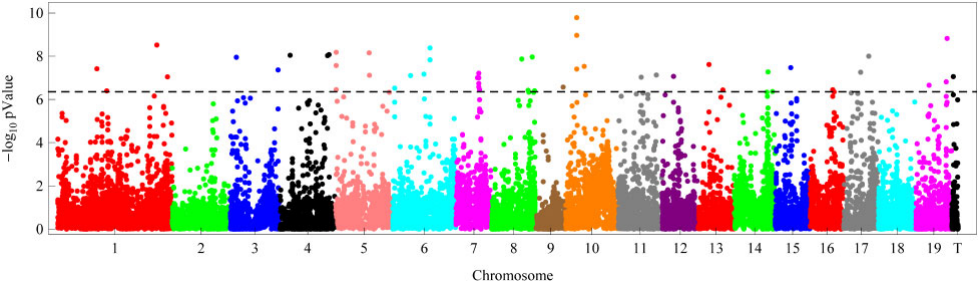
Manhattan plot of genome-wide association analysis of tree height in the randomized complete block experiment. The horizontal line indicates the genome-wide significant threshold of 6.36, a base 10 logarithm of *P-*value based on the Bonferroni correction at the 0.01 significant level.

We applied the mvLMM to perform the GWAS for the tree height with the 22,670 HQ SNPs distributed on the 19 chromosomes and several scaffolds in *Populus*. The *P*-value threshold for significant SNPs was set to 4.41E–7 (-log_10_(*P*-value) = 6.36) based on the Bonferroni correction at the 0.01 significant level. If a small region (<1000 bp) harbored several significant SNPs, the most significant was chosen to represent that region. As a result, 41 SNPs were found to be significantly associated with the tree height. Figure 2 shows the Manhattan plot of the SNP position against the corresponding negative base 10 logarithm of *P*-value. It can be seen that these significant SNPs were distributed on all but 2 (Chr02 and Chr18) chromosomes with one SNP on scaffold 45. The physical distance between adjacent significant SNPs was greater than 123 kb, the minimum distance found between the second and the third significant SNPs on chromosome 5 (Table 2).

**Table 2:** Summary of the significant SNPs associated with the tree height on chromosomes and scaffolds

SNP ID	Chromosome	Position	Segregation type	**-log_10_(** *P* **-value)**	*R* ^2^ (%)
DSC01H1^*^	Chr01	17015054	*ab*×*aa*	7.42	1.17
DSC01H2	Chr01	21306230	*aa*×*ab*	6.40	1.70
DSC01H3^*^	Chr01	43104197	*aa*×*ab*	8.52	2.43
DSC01H4	Chr01	47744299	*aa*×*ab*	7.04	1.47
DSC03H1	Chr03	2674990	*ab*×*aa*	7.95	1.02
DSC03H2	Chr03	20842084	*aa*×*ab*	7.37	2.34
DSC04H1^*^	Chr04	4423234	*aa*×*ab*	8.04	1.84
DSC04H2	Chr04	20708892	*aa*×*ab*	8.02	1.59
DSC04H3	Chr04	21344054	*ab*×*aa*	8.07	2.39
DSC05H1^*^	Chr05	336929	*aa*×*ab*	8.18	0.26
DSC05H2^*^	Chr05	14685227	*aa*×*ab*	8.15	1.84
DSC05H3^*^	Chr05	14808652	*ab*×*aa*	7.12	1.99
DSC06H1	Chr06	810179	*aa*×*ab*	6.52	1.35
DSC06H2	Chr06	7733888	*aa*×*ab*	7.10	1.19
DSC06H3^*^	Chr06	13575979	*ab*×*aa*	7.17	1.58
DSC06H4^*^	Chr06	16203991	*aa*×*ab*	8.38	1.13
DSC07H1	Chr07	9357528	*ab*×*aa*	7.00	1.60
DSC07H2	Chr07	9920573	*aa*×*ab*	7.21	2.38
DSC07H3^*^	Chr07	10256748	*aa*×*ab*	6.45	0.64
DSC08H1	Chr08	13071948	*ab*×*aa*	7.87	1.54
DSC08H2	Chr08	15844780	*ab*×*aa*	6.42	1.47
DSC08H3	Chr08	17642966	*aa*×*ab*	7.96	2.05
DSC08H4	Chr08	18644603	*ab*×*aa*	6.40	1.22
DSC09H1^*^	Chr09	11900120	*ab*×*aa*	6.57	1.48
DSC10H1^*^	Chr10	4825843	*aa*×*ab*	9.79	1.96
DSC10H2^*^	Chr10	8084409	*ab*×*aa*	7.53	0.58
DSC11H1	Chr11	10094625	*aa*×*ab*	7.03	0.46
DSC11H2	Chr11	16687705	*aa*×*ab*	7.13	1.97
DSC12H1	Chr12	4890486	*ab*×*aa*	7.07	1.33
DSC13H1^*^	Chr13	4732990	*ab*×*aa*	7.62	1.99
DSC13H2	Chr13	10764896	*aa*×*ab*	6.44	1.72
DSC14H1^*^	Chr14	14722576	*aa*×*ab*	7.27	1.22
DSC14H2	Chr14	16676502	*aa*×*ab*	6.36	1.99
DSC15H1	Chr15	6813100	*ab*×*aa*	7.47	1.37
DSC16H1^*^	Chr16	9864836	*ab*×*aa*	6.45	1.11
DSC17H1	Chr17	7378390	*ab*×*aa*	7.26	1.09
DSC17H2	Chr17	10908093	*ab*×*aa*	8.00	2.01
DSC19H1	Chr19	5741825	*aa*×*ab*	6.66	1.65
DSC19H2	Chr19	13202165	*ab*×*aa*	6.81	0.99
DSC19H3	Chr19	13578941	*ab*×*aa*	8.82	2.64
DST45H1	scaffold_45	28115	*ab*×*aa*	7.06	1.93

* Consistent significant SNPs in position with QTLs of tree height in *Populus* identified in previous studies.

**Figure 3:**
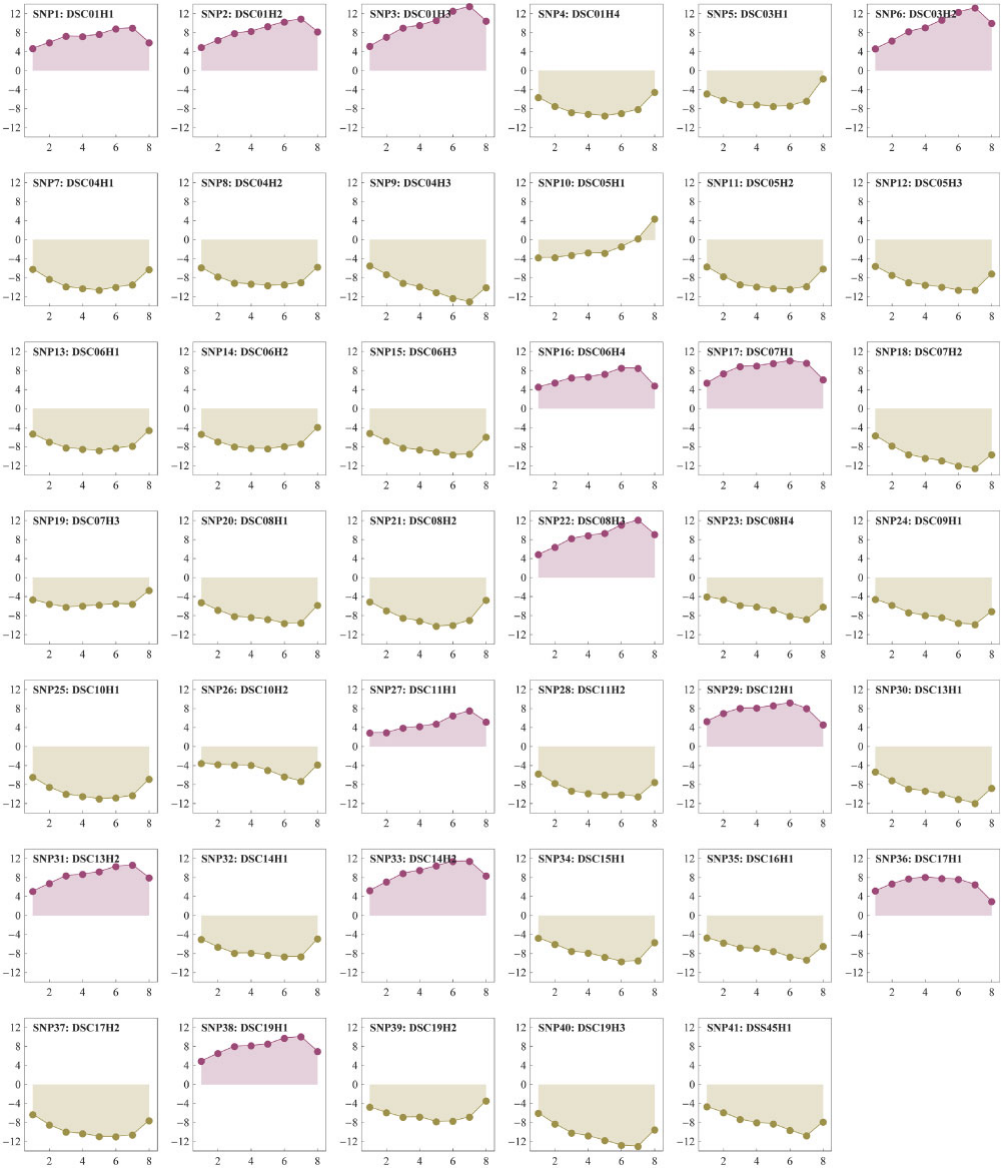
Scatter plots of SNP effects over the eight time points. Positive effect plots are labeled with purple and negative effect plots with light yellow.

Table 2 summarizes the significant SNPs on their IDs, positions, segregation types, *P*-values, and ratios of explaining the phenotypic variance (*R*^2^). The SNP IDs were named after the two parents of the clones currently used in this study, the chromosome and scaffold name, and the order within a chromosome, where D stands for *P. deltoides*, S for *P. simonii*, C for chromosome, and T for scaffold (*e.g.*, DSC05H2 indicates the second significant SNP affecting the tree height on chromosome 5). We found that all the SNPs segregated in the ratio of 1:1 with 21 segregating in the type of *aa*×*ab* and 20 in *ab*×*aa*. The percent of phenotypic variance (*R*^2^) explained by the SNPs ranged from 0.26% to 2.64%, amounting to 63.68% in total. Moreover, the effect of each SNP at each time point was also estimated, which was defined as the difference between the homozygous (*aa*) and heterozygous (*ab*) effects (Zeng 1994). Figure 3 presents the connected scatter plot of each SNP effects. It can be seen that the SNPs were largely divided into two categories: one possessed positive effects and the other had negative effects over multiple time points.

We found 15 significant SNPs were consistent in position with most QTLs identified in the previous studies for mapping tree height in *Populus* (Monclus *et al.* 2012; Du *et al.* 2016; Liu *et al.* 2017). Supplementary Table S7 lists those SNPs that were consistent with one or more QTLs detected in the three recent studies, excluding the two early studies due to no position information available in the physical map for the QTLs (Bradshaw and Stettler 1995; Wu 1998). It can be seen that most QTLs detected in the three previous studies either were located not far from or their confidence intervals contained a significant SNP. The physical distance between the consistent SNP and QTL was less than 5.0 Mb for most pairs with a few greater than 5.0 Mb but less than 15.0 Mb. Interestingly, we found that these consistent SNPs have stronger association signals than the others. It is obvious to see that 5 of the consistent SNPs have the first and the third to sixth lowest *P*-values (Table 2). Moreover, we performed the Kruskal–Wallis rank-sum test (Hollander and Wolfe 1973) to test the difference of the minus logarithm *P*-values between the 15 consistent SNPs and the others. The test result showed that the consistent SNP group had the minus logarithm *P*-values significantly higher than the other group with a *P*-value of 0.0482.

In comparison with the univariate approach, we also performed the association analysis for each single tree height with the software TASSEL. The result showed that there were 6 SNPs significantly associated with the tree height measured at the first time point (T1), which were distributed on chromosomes 4, 10, 17, and 19 (Supplementary Figure S1). However, no significant SNPs were found for the tree height at time points T2–T8.

### Exploration of candidate genes

**Figure 4:**
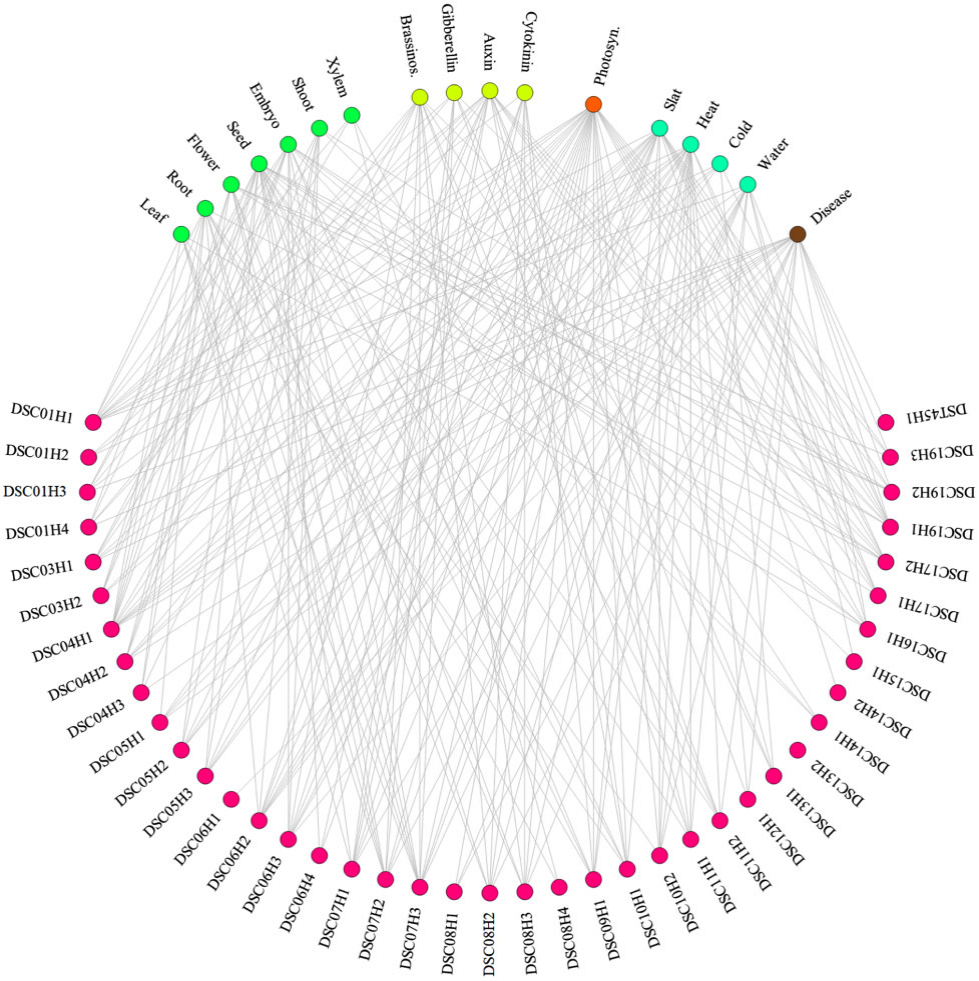
Significant SNPs with potential candidate genes related to biological functions and processes. All but one SNP (DS13H2) possessed candidate genes related to the tree growth and development of leaf, root, flower, seed, embryo, shoot, and xylem, to stress responses of salt, heat, cold, and water deprivation, and to disease resistance, or involved in brassinosteroid, gibberellin, auxin, cytokinin, and photosynthesis.

To explored candidate genes of the significant SNPs, we extracted 100 coding genes surrounding each SNP in the genome annotation database of *P. trichocarpa* at Phytozome (v4.1; https://genome.jgi.doe.gov). To obtain enough annotation information, the coding sequences were first blasted against the non-redundant (nr) protein database (Pruitt *et al.* 2007) and then the blast hits were mapped to Gene Ontology (GO; http://geneontology.org) terms. With the newly annotation result, we searched keywords related to tree growth and development, such as “brassinosteroid,” “gibberellin,” “leaf,” “xylem,” “photosynthesis,” “salt,” and “disease.” Consequently, 248 genes nearby the significant SNPs corresponded to at least one of the 17 keywords; all but one SNP (DSC13H2) had at least one candidate genes related to these words (Supplementary Table S8; Figure 4). It can be seen that there were 13, 8, 17, and 8 SNPs that had candidate genes involved in brassinosteroids, gibberellins, auxins, and cytokinins, respectively. These hormones were confirmed to have direct effects on plant height (Dubouzet *et al.* 2013). Furthermore, 7 SNPs possessed candidate genes related to leaf growth and development, 12 to root, 10 to flower, 21 to seed, 14 to embryo, 8 to shoot, and 4 to xylem. For responses to stress, 20 SNPs owned candidate genes for salt stress, 19 for heat stress, 6 for cold stress, and 12 for water deprivation or activity. It was surprising that up to 30 SNPs were identified with candidate genes related to photosynthesis, which plays an important role in the tree growth and development (Wang *et al.* 2017). In addition, 20 SNPs were found to be associated with candidate genes for disease resistance. Particularly, for the 15 significant SNPs consistent with the previous identified QTLs (Supplementary Table S7), we observed that all but one SNP (DSC05H1) had candidate genes that response to hormones or involve hormone activities, that all but two (DSC10H2 and DSC13H1) directly affected the growth and development of different tissues such as leaf, root, seed, and xylem, that all but three (DSC10H1, DSC10H2, and DSC14H1) were related to response to stresses or resistance to diseases, and that all but four (DSC05H1, DSC07H3, DSC10H2, and DSC14H1) were involved in photosynthesis.

To further understand the function of the 248 candidate genes described above, we conducted the GO enrichment analysis using all the 34,698 genes in the annotation database of *P*. *trichocarpa* (v4.1) as a reference set. As a result, 289 GO terms were significantly enriched with the FDR value <0.05, of which 223 belonged to the category of biological process, 40 to molecular function, and 26 to cellular component (Supplementary Table S9). Interestingly, almost all the searched words except “disease” were contained in at least one significant GO terms in the category of biological process (Table 3). For the four hormones, these GO terms included “response to brassinosteroid” (GO:0009741), “response to gibberellin” (GO:0009739), “response to auxin” (GO:0009733), and “response to cytokinin” (GO:0009735). For the tree tissues, the significantly enriched GO terms contained the developments of leaf (GO:0048366), root (GO:0048364), flower (GO:0009908), seed (GO:0048316), embryo (GO:0009790), shoot (GO:0048367), and xylem (GO:0010089). For the responses to stress, the similar GO terms consisted of responses to salt stress (GO:0009651), to heat (GO:0009408), to cold (GO:0009409), and to water deprivation (GO:0009414). Also, we found that the most enriched GO terms in Table 3 was “photosynthesis” (GO:0015979) with the smallest FDR value of 7.48E-24. Although no significant GO terms were found to be related to disease, there existed several significantly enriched descriptions related to disease resistance when we used annotations resulted from the blast hits in the enrichment analysis with Blast2Go (Supplementary Table S10). The reason that no significant GO terms were related to disease in this study may be due to the fact that there are only 2 GO terms (GO:0009614, GO:0106093) related to disease in the current GO database (http://geneontology.org).

**Table 3:** Some significantly enriched GO terms related to plant hormones, to the development of tree tissues, and to response to stresses, or involved in photosynthesis

GO ID	GO Name	FDR	*P* **-value**	Nr test	Nr reference	Non Annot test	Non Annot reference
GO:0009741	Response to brassinosteroid	1.1E–8	9.86E–11	9	42	239	34408
GO:0009742	Brassinosteroid mediated signaling pathway	4.91E–5	7.06E–7	6	38	242	34412
GO:0016131	Brassinosteroid metabolic process	0.0121	3.28E–4	4	42	244	34408
GO:0009739	Response to gibberellin	1.28E–4	1.94E–6	6	46	242	34404
GO:0009733	Response to auxin	2.69E–15	1.22E–17	22	229	226	34221
GO:0009734	Auxin-activated signaling pathway	6.84E–9	5.95E–11	12	106	236	34344
GO:0009735	Response to cytokinin	8.76E–7	9.15E–9	7	31	241	34419
GO:0009736	Cytokinin-activated signaling pathway	1.76E–6	1.98E–8	6	19	242	34431
GO:0009690	Cytokinin metabolic process	0.0039	8.86E–5	4	29	244	34421
GO:0048366	Leaf development	0.021	6.15E–4	5	90	243	34360
GO:0009965	Leaf morphogenesis	0.037	0.0011	3	26	245	34424
GO:0048364	Root development	6.87E–4	1.24E–5	7	100	241	34350
GO:0022622	Root system development	6.87E–4	1.24E–5	7	100	241	34350
GO:0009908	Flower development	0.0129	3.54E–4	7	175	241	34275
GO:0048316	Seed development	4.45E–10	3.41E–12	16	195	232	34255
GO:0090351	Seedling development	0.0064	1.55E–4	4	34	244	34416
GO:0009845	Seed germination	0.0468	0.0015	3	29	245	34421
GO:0009790	Embryo development	3.18E–5	4.09E–7	9	120	239	34330
GO:0048367	Shoot system development	1.81E–9	1.55E–11	18	295	230	34155
GO:0010016	Shoot system morphogenesis	2.15E–6	2.44E–8	8	58	240	34392
GO:0010089	Xylem development	0.009	2.28E–4	3	14	245	34436
GO:0010087	Phloem or xylem histogenesis	0.0121	3.28E–4	4	42	244	34408
GO:0015979	Photosynthesis	7.48E–24	7.81E–27	29	213	219	34237
GO:0009651	Response to salt stress	1.99E–22	3.46E–25	24	130	224	34320
GO:0009408	Response to heat	1.78E–13	1.01E–15	16	110	232	34340
GO:0009409	Response to cold	0.0172	4.8E–4	5	85	243	34365
GO:0009414	Response to water deprivation	1.01E–6	1.08E–8	9	76	239	34374

We also performed GO enrichment analyses to investigate the difference of candidate gene functions between the significant SNP group with positive effects and the group with negative effects (Figure 3). There were 71 and 177 candidate genes for the positive- and negative-effect groups, respectively. Two GO enrichment analyses were conducted with the 71 and 177 candidate genes as test sets separately and all the 34,698 genes as the reference set. The results showed that 95 GO terms were significantly enriched for the positive group and 220 for the negative group (Supplementary Tables S11 and S12). We observed that there were 73 GO terms enriched in both groups, such as “response to hormone,” “photosynthesis,” and “post-embryonic development.” However, there were 22 unique GO terms enriched in the positive group and up to 147 in the negative group. In the positive group, the unique enriched GO terms included “auxin-activated signaling pathway,” “stigma development,” “flower development,” and the others (Supplementary Table S11), whereas in the negative group those unique GO terms included “response to brassinosteroid,” “shoot system development,” “xylem development,” etc. (Supplementary Table S12). Although the candidate genes were involved in many GO terms, these unique GO terms could be used to link the two groups of significant SNPs.

## Discussion

### Application of traditional experimental design for GWAS

The RCBD is one of the most widely used experimental designs in traditional forest breeding program for estimating genetic parameters (Wright 1976; Williams *et al.* 2002). However, this kind of test experiments was rarely directly used with mvLMM to identify the associations between molecular markers and phenotypic traits, such as GWAS and QTL mapping, possibly due to the lack of suitable software. In this study, we first used mvLMM to perform GWAS of tree height with longitudinal measurements from the traditional RCBD in *Populus*. The poplar hybrid F_1_ clones were planted repeatedly not only among blocks but also within plots in the RCBD. One advantage of these repeated clones is to allow us to obtain repeated phenotypic data for a genotype that originated from a seed. Theoretically, the repeated data can control spatial effects in field and reduce the systematic errors, thus improving the accuracy and power in GWAS. Another advantage is that each genotype can be preserved almost forever especially in forest trees because the repeated plants provide redundancy for the same genotype in the case of natural damages caused by insect, disease or wind. On the contrary, in most previous GWAS or QTL mapping studies in *Populus* (Bradshaw and Stettler 1995; Monclus *et al.* 2012; Du *et al.* 2016; Liu *et al.* 2018), phenotypic traits were measured from single plants with different genotypes in natural populations or a full-sib family such as the traditional BC and F_2_ populations in inbred lines and the F_1_ hybrid population in outbred species. In these populations, each tree corresponds to a unique genotype so that either the number of genotypes could gradually reduce over times or distortion measurements could be produced on some genotypes owing to the natural damages. Such reduction in genotype number or existence of distortion measurements would greatly affect the genetic parameter estimation and thus discount the power of GWAS or QTL mapping. Overall, the RCBD established with poplar clones in this study provided a unique population for effectively studying the molecular mechanism underlying important traits in *Populus.*

### Implementation of GWAS with longitudinal measurements from the RCBD

We successfully applied mvLMM to the GWAS of tree height with longitudinal measurements from the RCBD in this study. Because of the unique population structure and the multiple phenotypic measurements, we cannot directly apply current available GWAS software to simultaneously estimate the genetic variance components of tree heights at the 8 time points. Generally, the software such as EMMA (Kang *et al.* 2008), EMMAX (Kang *et al.* 2010), and GEMMA (Zhou and Stephens 2012) are usually applied in animal and human GWAS by incorporating the minor allele effect of a bi-allelic SNP into LMM, in which only one column with elements of 0, 1, and 2 for SNP genotype effects is added in the design matrix of fixed effects. However, there were triallelic SNPs in our SNP data set (Table 1), which segregated in 1:1 (*aa*×*bc* and *ab*×*cc*), 1:2:1 (*ab*×*bb*), or 1:1:1:1 (*ab*×*ac*). Apparently, these SNPs cannot be applied into the GWAS software only for biallelic SNPs. To solve this problem, we used SNP genotype effects of each SNP in the LMM (1) with the restriction of the sum of expected genotype effects equal to zero. Next, the function emmremlMultivariate in the R package EMMREML was used to calculate the estimates of the genetic and residual matrices of variances. This function is flexible for the design matrix of the fixed effects and focuses on dealing with multivariate phenotypic traits, but it cannot flexibly provide statistics for testing SNPs if their genotype effects correspond to more than two columns in the design matrix. We resolved the hypothesis testing problem by converting the multivariate LMM (1) into univariate LMM through matrix vectorization (Searle *et al.* 2006) (pp. 458) and then conducting the hypothesis test using formula (3) as constructed in Kang *et al.* (Kang *et al.* 2008).

### The issue of additive genetic relationship matrix

One of the crucial parts in the LMMs is the additive genetic relationship matrix which reflects population structure and directly affects the estimate of background genetic variance. The relationship matrix is the kinship matrix multiplied by 2 (Lynch and Walsh 1998; Bae *et al.* 2016), which can be inferred by pedigree- or marker-based methods (Thornton and McPeek 2007; VanRaden 2008; Yang *et al.* 2010). Although various marker-based methods for inferring a kinship matrix with a large number of SNPs have been proposed, they showed small differences in correcting population structure (Nievergelt *et al.* 2007) and some of them had the limitation that the estimated kinship matrix may not be guaranteed to be positive semidefinite, which could distort the genetic parameter estimations (Kang *et al.* 2008). Moreover, these marker-based methods were typically based on the markers each segregating in three genotypes, that is, the homozygote, heterozygote, and other homozygote. The genotype effects were usually assumed to be a scale of 1, 0, and -1 with frequencies of $p_{i}^{2}$, $2p_{i}(1-p_{i})$, and ${\left(1-p_{i}\right)}^{2}$, respectively. The variance of the genotype effects can be derived as a scale of$2p_{i}(1-p_{i})$ and it was used to generate the relationship matrix (VanRaden 2008). However, in this study, the majority of SNPs are segregated only in two genotypes expressed as *aa* and *ab* with frequencies of $p_{i}$ and $1-p_{i}$ (Table 1), which is attributed to the two highly heterozygous parents of the F_1_ hybrid population (Mousavi *et al.* 2016; Tong *et al.* 2016). Apparently, the variance of the two genotype effects is totally different from that of the three genotype effects. Therefore, the relationship matrix cannot be properly calculated from our SNP data with currently available software such as TASSEL and EMMA. Nevertheless, we noted that our samples were all from a full-sib family of two unrelated parents in *Populus*. In theory, the coefficient of kinship (or coancestry) is expected to be 0.25 for full-sibs assuming random mating (Loiselle *et al.* 1995; Lynch and Walsh 1998). This led to the relationship matrix with elements of ones on the diagonal and 0.5 elsewhere, which was applied as a pedigree-method in this study.

### *P*-value threshold and the number of significant SNPs

The LMM is one of the most popular approaches in GWAS, but it tests one SNP at a time so as unable to simultaneously identify many loci that underlie the objective trait. For this reason, it is critical to determine the *P-*value threshold for significant SNPs by performing multiple testing. The most commonly used methods for multiple hypothesis testing include the Bonferroni correction and Benjamini–Hochberg (BH) procedure (Benjamini and Hochberg 1995). The Bonferroni correction is considered to be more conservative and certainly to result in less significant SNPs than the BH method. Both methods limit the false positive rate, but they very likely inflate the false-negative rate. We used the Bonferroni correction to determine the *P*-value threshold under a lower common significant level of 0.01, leading to 41 significant SNPs detected for the tree height. This means that the expected number of false-positive SNPs was less than one, but the number of negative false SNPs was uncertain. Such a medium number of extremely significant SNPs each explaining a small fraction of the phenotypic variance (0.26%–2.64%) is in accord with the infinitesimal model, assuming that a quantitative trait is typically controlled by an infinite number of genes each with a tiny effect (Fisher 1919; Bulmer 1971). Considering those cases of up to 100 significant SNPs found for a complex trait, such as in animal and crop GWAS (Ober *et al.* 2012; Bali *et al.* 2018), it is very likely that there exist a lot of undetected SNPs that have much smaller effects and explain the rest portion of the phenotypic variance in this study. However, we virtually identified far many more loci associated with tree height than QTLs detected in the previous QTL mapping studies in *Populus* (Bradshaw and Stettler 1995; Wu 1998; Monclus *et al.* 2012; Du *et al.* 2016; Liu *et al.* 2017). This could be attributed to the application of the traditional RCBD and the use of longitudinal phenotypic measurements in the LMM, enhancing the power of detecting association of SNPs to the tree height.

### Alternative approach for finding candidate genes

We used the nearby genes for each significant SNP to investigate candidate genes that could be related to the tree height under study. The genes nearby each SNP spanned a physical region with a mean length of 2.03 Mb and a standard deviation of 1.17 Mb. This approach could be called the proximate strategy, which has been frequently used for finding candidate genes in GWAS or QTL studies (Monclus *et al.* 2012; Geng *et al.* 2015; Su *et al.* 2017; Vanous *et al.* 2018). Alternatively, LD analysis can be used to search candidate genes with procedures as described by (Slaten *et al.* 2020). As a comparison, we also tried this method to find candidate genes that are in LD with the significant SNP. First, LD threshold was determined by performing LD decay analysis with all the SNPs using the software package PopLDdecay (Zhang *et al.* 2019). The resut showed that LD decayed rapidly to about $r^{2}=0.2$ corresponding to a physical distance of ∼650 bp (Supplementary Figure S2), which was consistent with the reports in the literature that LD decayed at a short distance of 100-1500 bp in outcrossing species and that the threshold of $r^{2}=0.2$ can be used at which LD stops to exist. Next, we used this threshold to find the candidate genes of a significant SNP on the condition that $r^{2}\geq 0.2$ between an SNP within a gene and this significant SNP. As a result, a total of 94 candidate genes were found for 31 significant SNPs, most (73.40%) of which were in low LD ($r^{2}<0.5$) with their corresponding significant SNPs (Supplementary Table S13). Unfortunately, few of these candidate genes had meaningful descriptions about the growth and development of tree height. This undesirable result could be explained by the fact that many genes around a significant SNP did not contain any SNPs due to the less number of SNPs available in this study as compared to the number of genes on the reference genome (22,670 versus 34,699), so that they had no chance to be chosen as candidate genes through LD analysis. Therefore, it was an appropriate way to use the proximate strategy for studying candidate genes of the significant SNPs in this study.

## Conclusion

The combined use of the traditional RCBD along longitudinal measurements could greatly improve the power of GWAS, leading to identifying far more significant SNPs associated with tree height than QTLs detected in previous studies in *Populus*. The detected SNPs were distributed on all but 2 chromosomes and explained a large portion of the phenotypic variance. Most of these SNPs possessed potential candidate genes that were significantly related to the growth and development of different tissues, to stress responses, and to disease resistance, or involved in several plant hormones. The result would enhance understanding of molecular mechanism for growth traits and would accelerate marker-assisted breeding programs in such species.

## Funding

This work was supported by the National Natural Science Foundation of China (Grant No. 31870654 and 31270706) awarded to CT and the Priority Academic Program Development of Jiangsu Higher Education Institutions.

*Conflicts of interest*: None declared.
